# Nuclear and kinetoplast DNA analyses reveal genetically complex *Leishmania* strains with hybrid and mito-nuclear discordance in Peru

**DOI:** 10.1371/journal.pntd.0008797

**Published:** 2020-10-19

**Authors:** Ahmed Tabbabi, Abraham G. Cáceres, T. Pershing Bustamante Chauca, Chisato Seki, Yanisa Choochartpong, Daiki Mizushima, Daisuke S. Yamamoto, Yoshihisa Hashiguchi, Hirotomo Kato

**Affiliations:** 1 Division of Medical Zoology, Department of Infection and Immunity, Jichi Medical University, Tochigi, Japan; 2 Sección de Entomología, Instituto de Medicina Tropical “Daniel A. Carrión” y Departamento Académico de Microbiología Médica, Facultad de Medicina Humana, Universidad Nacional Mayor de San Marcos, Lima, Perú; 3 Laboratorio de Entomología, Instituto Nacional de Salud, Lima, Perú; 4 Área de Investigación en Salud—Evaluación de las Intervenciones Sanitarias y Vigilancia de las Enfermedades Metaxénicas y Zoonoticas, Dirección Regional de Salud Amazonas, Perú; 5 Faculty of Veterinary Medicine, Khon Kaen University, Khon Kaen, Thailand; 6 Department of Parasitology, Kochi Medical School, Kochi University, Kochi, Japan; Institut Pasteur de Tunis, TUNISIA

## Abstract

Polymerase chain reaction-restriction fragment length polymorphism (PCR-RFLP) analysis of the mannose phosphate isomerase (*mpi*) gene was applied to 134 skin samples collected from patients with cutaneous leishmaniasis (CL) in Peru for identification of the infecting parasite at the species level, and the results were compared with those of cytochrome *b* (*cyt* b) gene sequencing obtained in previous studies. Although most results (121/134) including 4 hybrids of *Leishmania (Viannia) braziliensis* and *L*. *(V*.*) peruviana* corresponded to those obtained in the previous study, PCR-RFLP analyses revealed the distribution of putative hybrid strains between *L*. *(V*.*) peruviana* and *L*. *(V*.*) lainsoni* in two samples, which has never been reported. Moreover, parasite strains showing discordance between kinetoplast and nuclear genes (kDNA and nDNA), so-called mito-nuclear discordance, were identified in 11 samples. Of these, six strains had the kDNAs of *L*. *(V*.*) braziliensis* or *L*. *(V*.*) peruviana* and nDNAs of *L*. *(V*.*) guyanensis*, and three strains had the kDNAs of *L*. *(V*.*) shawi* and nDNAs of *L*. *(V*.*) braziliensis*. The rest were identified as mito-nuclear discordance strains having kDNAs of *L*. *(V*.*) braziliensis* or *L*. *(V*.*) peruviana* and nDNAs of *L*. *(V*.*) lainsoni*, and kDNAs of *L*. *(V*.*) lainsoni* and nDNAs of *L*. *(V*.*) braziliensis*. The results demonstrate that *Leishmania* strains in Peru are genetically more complex than previously considered.

## Author summary

Protozoan parasites of the genus *Leishmania* are able to undergo genetic exchange during their growth. The previous description of hybrids in Peru and the recent discovery of unexpected genetically complex strains having characteristics of both hybrid and mito-nuclear discordance in its neighbouring country (Ecuador) with a similar eco-epidemiological situation led us to consider that the genetic structure of *Leishmania* strains in Peru is more complicated than previously thought. In an effort to revise the data on *Leishmania* strain dispersion in Peru and to search for evidence of genetic recombination, the present study was conducted. A polymerase chain reaction-restriction fragment length polymorphism (PCR-RFLP) analysis targeting the mannose phosphate isomerase (*mpi*) gene sequence was performed to identify the infecting parasite at the species level in 134 skin samples collected from patients with cutaneous leishmaniasis (CL) in Peru, and the results were compared with those of cytochrome *b* (*cyt* b) gene sequencing obtained in previous studies. Most results (121/134) including 4 hybrids between *L*. *(V*.*) braziliensis* and *L*. *(V*.*) peruviana* showed agreement between PCR-RFLP of the *mpi* gene and *cyt* b gene sequence analysis; however, 13 of 134 samples revealed the distribution of strains with hybrids and mito-nuclear discordance. The results demonstrate that genetically complex *Leishmania* strains are present in Peru. These findings indicate that *Leishmania* strain dispersion in Peru is genetically more complex than previously considered. Further prospective studies including larger samples and the isolation of parasite strains are required to update the available data.

## Introduction

Parasitic protozoa belonging to the genus *Leishmania* show marked epidemiologic and clinical diversity, causing a wide-range of human and animal diseases extending from localized, self-limiting cutaneous lesions, and severe diffuse and destructive mucocutaneous lesions, to disseminating visceral infection that is fatal in the absence of treatment. The clinical heterogeneity, together with reservoir host spectrum and vector species compatibilities, lead to different parasite species associations, with over 20 species related to human infections. The cause of this heterogeneity, whether by continuous accumulation of mutations via mitotic cell division and/or via sexual recombination promoting admixtures of divergent genomes, is still a matter of debate [1]. Even if we are far from explaining how *Leishmania* parasites evolve and emerge in natural populations, correct species identification and searching for evidence of genetic recombination can provide key clues to the ecology and transmission patterns of these organisms. Correct diagnosis of leishmaniasis will also help to determine the clinical prognosis and an appropriate species-specific therapeutic regimen [2].

Multi-locus enzyme electrophoresis (MLEE) has been the gold standard for species characterization [3, 4]. However, this classification has been challenged based on genetic analysis of molecular targets, as they were much simpler, easier, and more practical [5–14]. Kinetoplast DNA (kDNA) is frequently used as a target for detection and typing of *Leishmania* due to its multicopy nature and high sensitivity [15, 16]. However, combining nuclear DNA (nDNA) and kDNA markers has improved the power of molecular data to detect unexpected genetically complex strains with characteristics of hybrid and mito-nuclear discordance widely distributed in Ecuador [17]. These recent findings have shown the necessity of updating the available data in its neighbouring country of Peru, with a similar eco-epidemiological situation, and searching for genetic recombination in Peruvian strains that were identified at the species level by kinetoplast cytochrome *b* (*cyt* b) gene sequence analysis in a previous study. The latter led to the identification of 7 species and 1 hybrid: *Leishmania (Viannia) braziliensis*, *L*. *(V*.*) peruviana*, *L*. *(V*.*) guyanensis*, *L*. *(V*.*) lainsoni*, *L*. *(V*.*) shawi*, *Leishmania (Leishmania) mexicana*, *L*. *(L*.*) amazonensis*, and a hybrid of *L*. *(V*.*) braziliensis/L*. *(V*.*) peruviana* [18]. Previous results on the current epidemiological situation in Peru showed that the predominant species were *L*. *(V*.*) peruviana*, *L*. *(V*.*) braziliensis*, and *L*. *(V*.*) guyanensis* in the Andean highlands, tropical lowland rainforests, and northern to central rainforest areas, respectively [19–22]. These studies also demonstrated the presence of *L*. *(V*.*) lainsoni* and *L*. *(L*.*) amazonensis* in lower-altitude rainforest areas [20–22], and the current prevalence in the same areas was confirmed [18]. The identification of hybrid forms was performed using a polymerase chain reaction-restriction fragment length polymorphism (PCR- RFLP) analysis of a short mannose phosphate isomerase (*mpi*) gene fragment. Hybrid genotypes have been rarely revealed among natural isolates of *Leishmania* parasites, which conventionally indicated that members of the genus possess the machinery for genetic exchange [17, 19]. A hybrid of *L*. *(V*.*) braziliensis* and *L*. *(V*.*) peruviana* was first reported in the Department of Huanuco [19, 23], and was recently identified in the Department of Cajamarca [18]. Additionally, just four cases of infection by *L*. *(V*.*) shawi* were detected in rainforest areas by the Departments of Junin, Madre de Dios, Cusco, and Puno, as previously reported [18, 22], and the question remains unanswered about the low rate of infection caused by this species. Verification of the full details of gene property variants will help to understand the eco-epidemiological situation in this endemic area.

The present study utilized PCR-RFLP analysis targeting the *mpi* gene sequence to identify the infecting parasite at the species level in 134 skin samples collected from patients with cutaneous leishmaniasis (CL) in Peru, and the results were compared with those of *cyt* b gene sequencing obtained in previous studies. The results demonstrate that genetically complex *Leishmania* strains are present in Peru, highlighting the need to combine both nDNA and kDNA targets to improve the validity of species identifications for full details of gene property variants.

## Materials and methods

### Sample collection

The clinical samples employed in this study were collected from patients suspected of having CL from 58 sites in 38 provinces administered by 17 departments in Peru using FTA cards (Whatman, Newton Center, MA, USA) and Giemsa-stained smears in previous studies [18, 22] (S1 Fig). Only identified specimens in previous studies were used in this study to avoid selection bias. The preparation of FTA Card samples was done as previously described [18]. Briefly, two-mm-diameter disks were punched out from each filter paper. The disks were then transferred to separate tubes, washed two times with FTA Purification Reagent (Whatman) and one time with Tris-EDTA buffer, and then air dried. The filter paper disks were directly subjected to PCR amplification. DNA extraction from Giemsa-stained smears obtained from skin lesions (ulcers and/or nodules) on CL patients was performed as previously described [18]. Briefly, 50 μL of DNA extraction buffer [150 mM NaCl, 10 mM Tris-HCl (pH 8.0), 10 mM EDTA, and 0.1% sodium dodecyl sulfate (SDS)] containing 100 μg/mL of proteinase K was spotted onto each smear sample and mixed well. The samples were transferred to 1.5-mL tubes and incubated at 37°C for 12 hours. After being heat-inactivated at 95°C for 5 min, 0.5-μL portions were directly used as the templates for PCR amplification.

### PCR and sequence analysis

Multiple targets including *mpi*, 6-phosphogluconate dehydrogenase (*6pgd*), heat shock protein 70 (*hsp70*), and cytochrome oxidase subunit II-NADH dehydrogenase subunit I (*COII-ND1*) gene fragments were used to confirm species identifications for full details of gene property variants. The primers and PCR conditions were described previously [17] except for the *mpi* gene fragment, where new primers were designed to amplify a shorter fragment (S2 and S3 Figs). For the *mpi* gene fragment, PCR amplification with a pair of specific primers, L.MPI-AS (5’- TCGATTCGCACGGCTCTGTC-3’) and L.MPI-OR (5’-CTCAAGTCGTTGGTCGACGC-3’), was performed with 30 cycles of denaturation (95°C, 1 min), annealing (55°C, 1 min), and polymerization (72°C, 1 min) using Ampdirect Plus reagent (Shimadzu Biotech, Tsukuba, Japan) and high-fidelity DNA polymerase, KOD plus (Toyobo, Osaka, Japan), or PrimeSTAR HS (Takara Bio, Shiga, Japan). Each 0.5-μL portion of the PCR product was reamplified with L.MPI-IR2 (5’-GCCGTACGGYACCGCAAAGC-3’) and L.MPI-BS (5’-AACCACAAGCCWGAGCTCAT-3’).

### PCR-RFLP analysis

PCR products of the *mpi* gene fragment were digested with the restriction enzymes *Hae*III, *Vpa*K11BI, and *Bst*XI. The restriction enzymes were selected with the support program BioEdit. The digested samples were separated by electrophoresis in 2% agarose gel (STAR Agarose RSV-AGRP from Rikaken, Aichi, Japan) for *Vpa*K11BI and *Bst*XI to separate longer fragments and 3% gel for *Hae*III to separate shorter fragments in order to produce DNA fragment patterns using the GeneRuler 100 bp Plus DNA ladder (Thermo Fisher Scientific, Waltham, MA, USA) as molecular size markers. The gel was stained with GelRed Nucleic Acid Gel Stain (Biotium, Hayward, CA, USA), and DNA fragments were visualized with a UV transilluminator.

### Ethics statement

Informed consent was obtained from all participating adults; for children under the age of 18 years, consent was also obtained from their parents or guardians. Verbal explanations and written information sheets were provided, following the guidelines of the Ethics Committee of the Ministry of Health, Peru. Clinical samples were collected by well-trained local doctors, biologists, and laboratory technicians from health posts and centers of the ministry. The subjects studied were volunteers in routine diagnosis/screening and treatment programs promoted by the ministry. All routine laboratory examinations were carried out free of charge, and treatment with a specific drug, sodium stibogluconate (Pentostan), was also offered free of charge at each health center. The study was approved by the ethics committee of the Graduate School of Veterinary Medicine, Hokkaido University (approval number: vet26-4) and Jichi Medical University (approval number: 17–080) [17, 18]. A case report form (CRF) was collected for each sample and unique codes were assigned to ensure confidentiality. All subjects and guardians consented to lesions being photographed anonymously. Permission was obtained from the Ministry of Health and from community leaders in Peru.

## Results

### Identification of *Leishmania* species using PCR-RFLP

**Fig 1:**
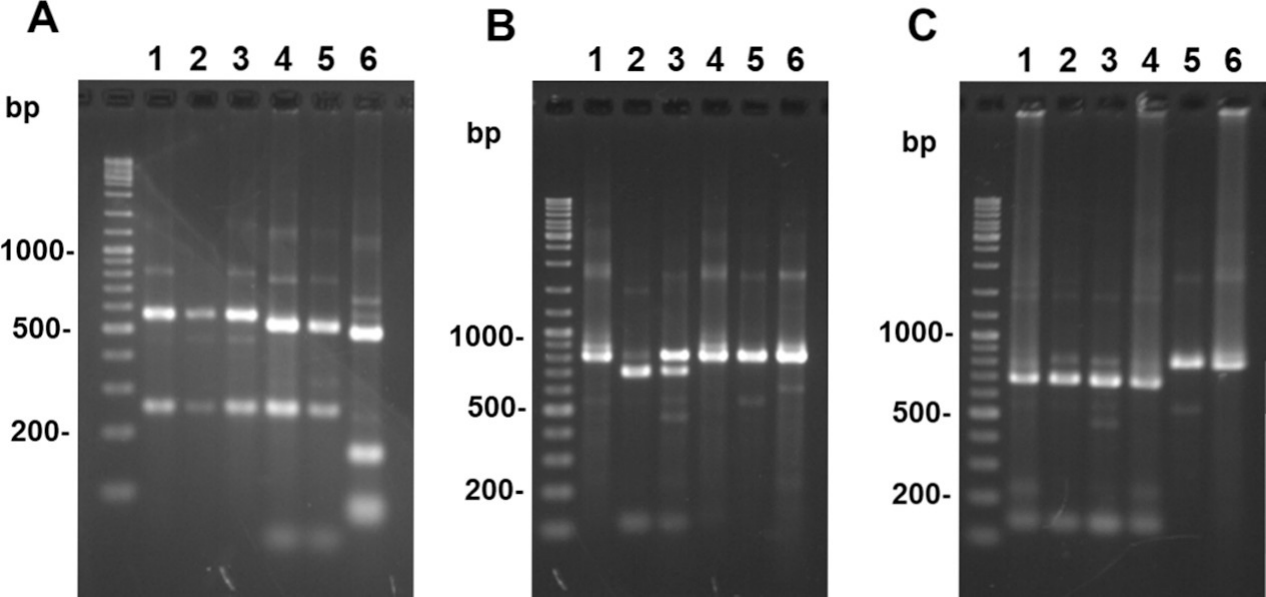
PCR-RFLP analyses of *mpi* gene fragments from 6 *Leishmania* species in Peru. PCR amplification was performed with leishmanial mannose phosphate isomerase (*mpi*) gene-specific primers, and PCR products were digested with *Hae*III (A), *Vpa*k11BI (B), or *Bst*XI (C), and resulting restriction fragment patterns were analyzed by agarose gel electrophoresis. 1. *L*. *(V*.*) braziliensis*, 2. *L*. *(V*.*) peruviana*, 3. a hybrid of *L*. *(V*.*) braziliensis/L*. *(V*.*) peruviana*, 4. *L*. *(V*.*) guyanensis*, 5. *L*. *(V*.*) lainsoni*, 6. *L*. *(L*.*) amazonensis*.

**Fig 2:**
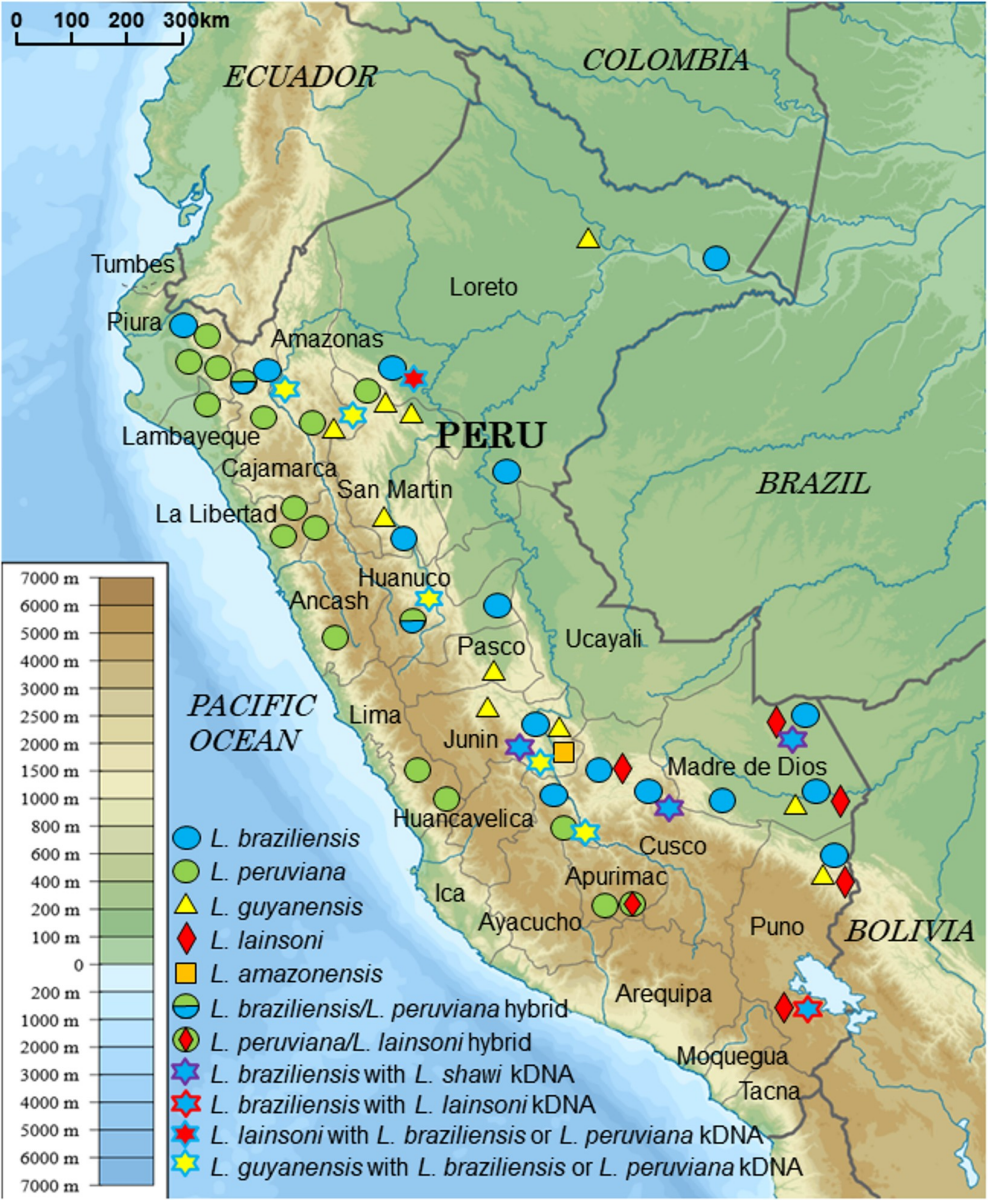
Geographic distribution of *Leishmania* species in Peru identified by PCR-RFLP of *mpi* and sequence analyses of kinetoplast and nuclear DNAs. (Adapted from a map available at https://commons.wikimedia.org/wiki/File%3APeru_physical_map.svg).

In a recent study, PCR-RFLP analyses of a long *mpi* gene fragment (1,130 bp) using the restriction enzymes *Hae*III and *Hpa*I differentiated *Leishmania* species in Ecuador, except for two very closely-related species, *L*. *(V*.*) guyanensis* and *L*. *(V*.*) panamensis* [17]. In addition, using a short *mpi* gene fragment (460 bp), 4 *Leishmania* species, *L*. *(V*.*) braziliensis*, *L*. *(V*.*) peruviana*, *L*. *(V*.*) guyanensis*, and *L*. *(V*.*) lainsoni*, and a hybrid of *L*. *(V*.*) braziliensis* and *L*. *(V*.*) peruviana*, could be differentiated in the Department of Huanuco, Peru [23]. In the present study, new primers were designed to amplify a 807 bp*-mpi* fragment (S2 Fig), and the PCR-RFLP technique was optimized using three diagnostic restriction enzymes (*Hae*III, *Vpa*K11BI, and *Bst*XI) for species identifications in the studied area. The RFLP patterns were predicted according to the sequences obtained from the GenBank database, and were tested on the *Leishmania* species listed in S1 Table (Fig 1). The application of PCR-RFLP to 134 skin samples collected from patients with CL in Peru (Fig 2) identified 5 *Leishmania* species [*L*. *(V*.*) braziliensis*, *L*. *(V*.*) peruviana*, *L*. *(V*.*) guyanensis*, *L*. *(V*.*) lainsoni*, and *L (L*.*) amazonensis*] and two putative hybrids [*L*. *(V*.*) braziliensis*/*L*. *(V*.*) peruviana* and *L*. *(V*.*) peruviana*/*L*. *(V*.*) lainsoni*] (Table 1). However, an unexpected RFLP pattern appeared in one sample, in which a single polymorphism nucleotide prevented the enzymes from cutting the target position, corresponding to *L*. *(V*.*) braziliensis* based on sequence analysis.

**Table 1:** Distribution of *Leishmania* species by department in Peru.

Department	*Leishmania* species*							Total
	Lb	Lp	Lg	Ll	La	Lb/Lp	Lp/Ll	
Amazonas	0	1	3 (2^#^)	0	0	0	0	4
Ancash	0	1	0	0	0	0	0	1
Apurimac	0	1	0	0	0	0	2	3
Ayacucho	2	1	2^#^	0	0	0	0	5
Cajamarca	1	1	0	0	0	3	0	5
Cusco	4 (1^#^)	0	0	1	0	0	0	5
Huanuco	3	0	1^#^	0	0	1	0	5
Junin	2 (1^#^)	0	3 (1^#^)	0	1	0	0	6
La Libertad	0	8	0	0	0	0	0	8
Lambayeque	0	18	0	0	0	0	0	18
Lima	0	2	0	0	0	0	0	2
Loreto	3	0	1	1^#^	0	0	0	5
Madre de Dios	24 (1^#^)	0	1	2	0	0	0	27
Pasco	0	0	2	0	0	0	0	2
Piura	1	10	0	0	0	0	0	11
Puno	6 (1^#^)	0	1	12	0	0	0	19
San Martin	1	1	6	0	0	0	0	8
Total	47	44	20	16	1	4	2	134

*Parasite species were identified by the PCR-RFLP analysis targeting 807bp *mpi* gene fragments (S2 Fig) with restriction enzymes *Hae*III, *Vpa*K11BI, and *Bst*XI. Lb, *L*. *(V*.*) braziliensis*; Lp, *L*. *(V*.*) peruviana*; Lg, *L*. *(V*.*) guyanensis*; Ll, *L*. *(V*.*) lainsoni*; *La*, *L (L*.*) amazonensis*; Lb/Lp, a putative hybrid of *L*. *(V*.*) braziliensis* and *L*. *(V*.*) peruviana*; Lb/Ll, a putative hybrid of *L*. *(V*.*) peruviana* and *L*. *(V*.*) lainsoni*

^#^Mito-nuclear discordance was determined by the comparative analysis of nuclear (*mpi*, *hsp70*, and *6pgd*) and kinetoplast (*cyt* b and *COII-ND1*) DNA fragments.

### Comparative analysis of nDNA and kDNA

**Fig 3:**
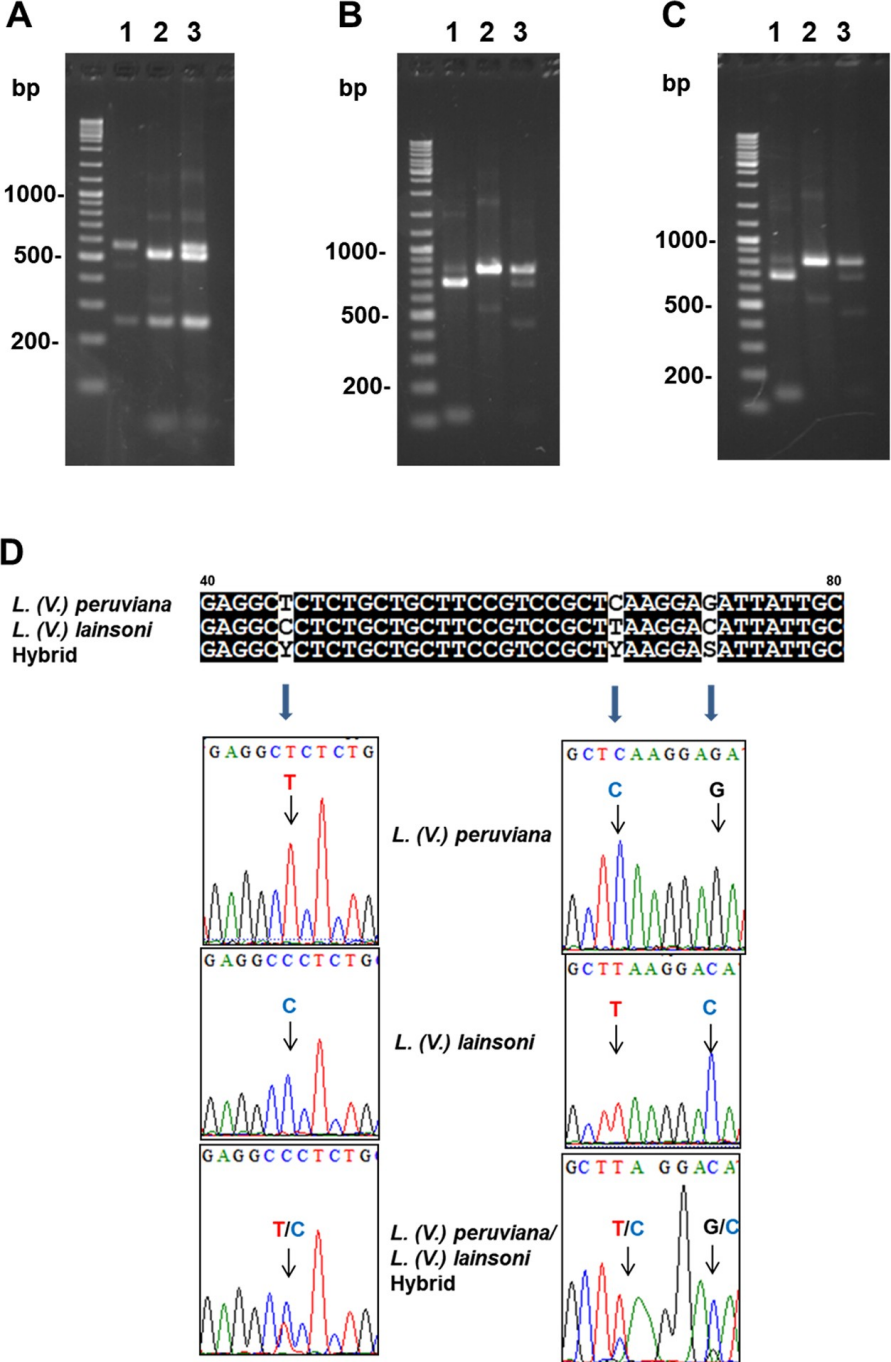
Differentiation between *L*. *(V*.*) peruviana* and *L*. *(V*.*) lainsoni* by PCR-RFLP and direct sequence analyses of *mpi* gene fragment. A, B, C. PCR amplification was performed with *mpi* gene-specific primers and the PCR products were digested with *Hae*III (A), *Vpa*k11BI (B), or *Bst*XI (C). 1. *L*. *(V*.*) peruviana*, 2. *L*. *(V*.*) lainsoni*, 3. a hybrid of *L*. *(V*.*) peruviana* and *L*. *(V*.*) lainsoni*. D. Direct sequence analysis showing three among several species-specific polymorphic sites of the *Leishmania mpi* gene fragment.

The results obtained by PCR-RFLP analyses were compared with those obtained by *cyt* b gene sequence analysis. In the previous studies, *L*. *(V*.*) braziliensis* was differentiated from *L*. *(V*.*) peruviana* by PCR-RFLP of the 460bp *mpi* gene fragment (S2 Fig) since the two species cannot be differentiated by kDNA sequence analysis [18,22]. Most results including 4 hybrids between *L*. *(V*.*) braziliensis* and *L*. *(V*.*) peruviana* agreed with previous ones obtained by *cyt* b gene sequence analysis; however, 13 of 134 samples showed discordance between the present and previous analyses (Tables 1 and 2) [18, 22]. In detail, of the 100 samples identified as *L*. *(V*.*) braziliensis* or *L*. *(V*.*) peruviana* by *cyt* b gene sequence analysis, 43, 44, and 4 samples were identified as *L*. *(V*.*) braziliensis*, *L*. *(V*.*) peruviana*, and a hybrid between *L*. *(V*.*) braziliensis* and *L*. *(V*.*) peruviana* by PCR-RFLP analyses of the *mpi* gene, corresponding to previous studies [18, 22]. On the other hand, six samples from wide areas of Peru and one sample from the north area, all of which were identified as *L*. *(V*.*) braziliensis* or *L*. *(V*.*) peruviana* by the *cyt* b gene sequence analysis, were *L*. *(V*.*) guyanensis* and *L*. *(V*.*) lainsoni*, respectively, by PCR-RFLP analyses of the *mpi* gene (Table 1). Sequence analyses of the *mpi* gene fragment supported the results of PCR-RFLP. In addition, two samples identified as *L*. *(V*.*) peruviana* using sequence analysis of the *cyt* b gene and PCR-RFLP analysis of the short *mpi* gene fragment in the previous studies showed hybrid patterns with *L*. *(V*.*) lainsoni* using PCR-RFLP analyses targeting 807bp-*mpi* gene fragments in the present study (Fig 3A–3C). Direct sequencing of the *mpi* fragments identified double peaks at the corresponding positions (Figs 3D and S3). According to our knowledge, this is the first reported evidence of putative hybridization between *L*. *(V*.*) peruviana* and *L*. *(V*.*) lainsoni*. On the other hand, of the 16 samples identified as *L*. *(V*.*) lainsoni* by *cyt* b gene analysis, 15 samples were identified as *L*. *(V*.*) lainsoni* whereas one sample was *L*. *(V*.*) braziliensis* by PCR-RFLP analyses of the *mpi* gene. In the same way, all three samples identified as *L*. *(V*.*) shawi* by sequence analysis of the *cyt* b gene were identified as *L*. *(V*.*) braziliensis* by PCR-RFLP analysis of the *mpi* gene. The mito-nuclear discordances having kDNA of *L*. *(V*.*) braziliensis* or *L*. *(V*.*) peruviana* and nDNA of *L*. *(V*.*) lainsoni*, and kDNA of *L*. *(V*.*) braziliensis* or *L*. *(V*.*) peruviana* and nDNA of *L*. *(V*.*) shawi* have not been reported in Peru or elsewhere. This is also the first reported evidence of mito-nuclear discordance having kDNA of *L*. *(V*.*) braziliensis* or *L*. *(V*.*) peruviana* and nDNA of *L*. *(V*.*) guyanensis* in Peru. Eleven samples showing discordance between *mpi* gene and *cyt* b gene analyses were systematically analyzed using multiple targets including *mpi*, *hsp70*, *6pgd*, and *COII-ND1* genes to confirm mito-nuclear discordance (Tables 1 and 2). Sequence analysis confirmed the discordance between nDNAs and kDNAs in these samples. The nucleotide sequence data reported were deposited in GenBank databases under the accession numbers LC517842-LC517879.

**Table 2:** Comparison of *Leishmania* species identification in Peru targeting kinetoplast and nuclear DNAs.

Target DNA^1)^	Identification^2)^: numbers						
kDNA	Lb or Lp^3)^: 100			Lg: 14	Ll: 16	Ls: 3	La: 1
nDNA	Lb: 43	Lp: 44	Lb/Lp: 4	Lg: 14	Ll: 15	Lb: 3	La: 1
	Lg: 6	Ll: 1	Lp/Ll: 2		Lb: 1		
kDNA + nDNA	Lb: 43	Lp: 44	Lb/Lp: 4	Lg: 14	Ll: 15	Ls-Lb: 3	La: 1
	Lb,p-Lg: 6	Lp/Ll: 2			Ll-Lb: 1		
	Lb,p-Ll: 1						

^1)^kDNA: kinetoplast DNA, nDNA: nuclear DNA. Parasite species were identified by the sequencing analysis of *cyt* b gene (kDNA) and by the PCR-RFLP analysis of *mpi* gene (nDNA) fragments with restriction enzymes *Hae*III, *Vpa*K11BI, and *Bst*XI. Mito-nuclear discordance was further determined by the comparative analysis of nDNA (*hsp70*, and *6pgd*) and kDNA (*COII-ND1*) fragments.

^2)^Lb: *L*. *(V*.*) braziliensis*, Lp: *L*. *(V*.*) peruviana*, Lg: *L*. *(V*.*) guyanensis*, Ll: *L*. *(V*.*) lainsoni*, Ls: *L*. *(V*.*) shawi*, La: *L*. *(L*.*) amazonensis*, Lb/Lp: a putative hybrid of *L*. *(V*.*) braziliensis* and *L*. *(V*.*) peruviana*, Lp/Ll: a putative hybrid of *L*. *(V*.*) peruviana* and *L*. *(V*.*) lainsoni*, Lb,p-Lg: *L*. *(V*.*) guyanensis* with kDNAs of *L*. *(V*.*) braziliensis* or *L*. *(V*.*) peruviana*, Lb,p-Ll: *L*. *(V*.*) lainsoni* with kDNAs of *L*. *(V*.*) braziliensis* or *L*. *(V*.*) peruviana*, Ll-Lb: *L*. *(V*.*) braziliensis* with *L*. *(V*.*) lainsoni* kDNA, Ls-Lb: *L*. *(V*.*) braziliensis* with *L*. *(V*.*) shawi* kDNA

^3)^*L*. *(V*.*) braziliensis* and *L*. *(V*.*) peruviana* cannot be differentiated by kDNA sequence analysis.

## Discussion

Comparative analysis of nuclear and kinetoplast DNA fragments revealed the occurrence of 5 *Leishmania* species in the study areas: *L*. *(V*.*) braziliensis*, *L*. *(V*.*) peruviana*, *L*. *(V*.*) guyanensis*, *L*. *(V*.*) lainsoni*, and *L*. *(L*.*) amazonensis*, and two putative hybrids: *L*. *(V*.*) braziliensis/L*. *(V*.*) peruviana* and *L*. *(V*.*) peruviana*/*L*. *(V*.*) lainsoni*, of which the latter was firstly identified in Peru. We also identified mito-nuclear discordances having kDNAs of *L*. *(V*.*) braziliensis* or *L*. *(V*.*) peruviana* and nDNAs of *L*. *(V*.*) lainsoni*, and kDNAs of *L*. *(V*.*) braziliensis* or *L*. *(V*.*) peruviana* and nDNAs of *L*. *(V*.*) shawi*, which have never been reported in Peru or elsewhere. In addition, we reported here the first evidence of mito-nuclear discordance having kDNAs of *L*. *(V*.*) braziliensis* or *L*. *(V*.*) peruviana* and nDNAs of *L*. *(V*.*) guyanensis* in Peru. Based on the geographic distribution, kDNAs of these mito-nuclear discordance strains probably originate from *L*. *(V*.*) braziliensis* since *L*. *(V*.*) peruviana* is prevalent mostly in Andean highland areas. Unexpectedly, approximately 10% of the samples were identified as strains with mito-nuclear discordance or hybrids caused by genetic exchange, indicating that the genetic structure of *Leishmania* strains in Peru is more complicated than previously recorded [18].

Of the 134 studied samples, 2 and 4 samples were identified as putative hybrids of *L*. *(V*.*) peruviana*/*L*. *(V*.*) lainsoni* and *L*. *(V*.*) braziliensis/L*. *(V*.*) peruviana*, respectively. Regarding its capacity to identify hybrids of these species, the PCR-RFLP method should be considered as a fundamental tool to be applied. Here, we report the first putative hybrid between *L*. *(V*.*) peruviana* and *L*. *(V*.*) lainsoni*. Natural hybrids were reported to occur episodically [17, 24–33]. In this context, it is important to note that most hybridization reported was either intra-specific or involved very closely related species: *L*. *(V*.*) braziliensis* and *L*. *(V*.*) peruviana*, although hybrids of more distant species have also been reported, such as *L*. *(L)*. *major/L*. *(L*.*) infantum* [33] and *L*. *(L*.*)* major/*L*. *(L*.*)* arabica [31]. The compatibility of different species to construct hybrids is unknown, and our finding of a putative *L*. *(V*.*) peruviana*/*L*. *(V*.*) lainsoni* hybrid expands the known natural compatibility between species. Both species are quite different with respect to these characteristics, and the impact of hybridization between such divergent species is currently unknown. Although the possibility of co-infection cannot be excluded completely, both alleles were proportionately amplified in PCR-RFLP analysis, strongly suggesting infection by hybrid strains in these patients. It is important to note here that isolation of putative hybrid strains as a culture is required for further detailed characterization of these parasites, since the possibility of co-infection cannot be excluded completely. *Lutzomyia ayacuchensis* is a proven vector of *L*. *(V*.*) peruviana* in the Andean area of southern Peru [34], whereas no transmission of *L*. *(V*.*) lainsoni* by sand fly species has been reported in these areas. Further investigation in relation to isolation of natural hybrids strains may be necessary for basic parasitological research on how and where genetic exchange occurs among *Leishmania* species. The genetic exchange may affect vector susceptibility, and hybrid strains may be transmitted by both vectors of parental species/strains [35]. In addition, using an animal model, hybrids between *L*. *(V.) braziliensis* and *L*. *(V.) peruviana* have been suggested to increase the disease severity when compared with the parental strains [36]. The pathological impact of recombinant genotypes in human infections associated with virulence, transmissibility, and drug susceptibility warrants further investigation in relation to improving methods of identification and isolation of natural hybrid strains.

Our results are the first reported evidence of mito-nuclear discordance among *Leishmania* species from Peru, highlighting the importance of combining both nDNA and kDNA to improve the validity of species identification for full details of gene property variants. Here, we report the first evidence of mito-nuclear discordance having kDNAs of *L*. *(V*.*) braziliensis* or *L*. *(V*.*) peruviana* and nDNAs of *L*. *(V*.*) guyanensis* in Peru, which was reported in Ecuador [17]. In this context, it is important to note that the most mito-nuclear discordant strains were found in the species identified as *L*. *(V*.*) braziliensis* or *L*. *(V*.*) peruviana* using *cyt* b gene analysis. The present study showed the occurrence of mito-nuclear discordance having kDNAs of *L*. *(V*.*) braziliensis* or *L*. *(V*.*) peruviana* and nDNAs of *L*. *(V*.*) shawi*, never detected in Peru or elsewhere. In this context, it is important to note that just four cases infected by *L*. *(V*.*) shawi* were detected in rainforest areas by the Departments of Junin, Madre de Dios, Cusco, and Puno, as reported previously [18, 22] and the question remains unanswered about the rarity of infection caused by this species. *L*. *(V*.*) shawi* was originally identified as a parasite of wild animals, and sand fly species that transmit it may prefer to feed on animals rather than humans, resulting in a steady rate of infections in wild animals via sand fly bites and a lower risk of infection for humans. However, further investigations using both and nDNA and kDNA genes may provide more clarifications about its epidemiological roles and geographical distributions including mito-nuclear discordance, as reported in the present work. Another unexpected finding was the identification of mito-nuclear discordant strains having kDNAs of *L*. *(V*.*) braziliensis* or *L*. *(V*.*) peruviana* and nDNAs of *L*. *(V*.*) lainsoni*, and kDNAs of *L*. *(V*.*) lainsoni* and nDNAs of *L*. *(V*.*) braziliensis*, never reported in Peru or elsewhere. Only one mito-nuclear discordant strain was identified in the species identified as *L*. *(V*.*) lainsoni* by *cyt* b gene analysis, which may confuse the previous results on identifications [18]. Further investigations are required to elucidate the mechanism of mito-nuclear discordance in protozoa. Several mechanisms have been shown to cause mito-nuclear discordance: incomplete lineage sorting, sex-biased asymmetries, introgression, natural selection, and genetic sweeps mediated by *Wolbachia* infection [37]. Mito-nuclear discordance was firstly reported in systems where early genetic tools were more developed compared with other taxa such us mitochondrial introgression between two species of fruit fly (*Drosophila pseudoobscura* and *D*. *persimilis*) and two species of mouse (*Mus domesticus* and *M*. *musculus*) [38, 39]. The number of cases continued to increase slowly until 2001. Following these early discoveries, methods for assaying numerous individuals for their nuclear genotype became more widely available to researchers and, subsequently, the number of cases increased markedly in animals such as mammals, birds, reptiles, amphibians, fish, and insects [37]. More recent studies revealed that mito-nuclear discordance occurs in helminth parasites [40–44].

In the present study, PCR- RFLP targeting leishmanial *mpi* gene fragments was performed, and the results were compared with those obtained by kinetoplast *cyt* b gene sequence analysis. The PCR-RFLP method was shown to be practical for the identification of *Leishmania* species in Peru, revealing unexpected genetically complex *Leishmania* strains with characteristics of hybrid and mito-nuclear discordance. Since hybrid strains were proposed to aggravate the severity of disease and may be transmitted by a larger range of sand fly species, many questions have been raised about the occurrence and frequency of such cross-species genetic exchange under natural conditions, modalities of hybrid transmission, their long-term maintenance, and the consequences of these transfers on phenotypes such as drug resistance or pathogenicity. Further country-wide epidemiological studies will be needed to reveal the characteristics of hybrid and mito-nuclear discordance and provide further insight into the mechanism of genetic exchanges of these parasites.

## Supporting information

S1 Fig The locations where one or more *Leishmania* specimens were collected. 1, Utcubamba, Amazonas; 2, Rodriguez de Mendoza, Amazonas; 3, Luya, Amazonas; 4, Huarmey, Ancash; 5, Aymaraes, Apurimac; 6, La Mar, Ayacucho; 7, Huanta, Ayacucho; 8, Jaen, Cajamarca; 9, Cutervo, Cajamarca; 10, Calca, Cusco; 11, La Convención, Cusco; 12, Puerto Inca, Huanuco; 13, Huanuco, Huanuco; 14, Leoncio Prado, Huanuco; 15, Chanchamayo, Junin; 16, Satipo, Junin; 17, Otuzco, La Libertad; 18, Santiago de Chuco, La Libertad; 19, Gran Chimú, La Libertad; 20, Lambayeque, Lambayeque; 21, Yauyos, Lima; 22, Huarochirí, Lima; 23, Alto Amazonas, Loreto; 24, Maynas, Loreto; 25, Mariscal Ramón Castilla, Loreto; 26, Ucayali, Loreto; 27, Tahuamanu, Madre de Dios; 28, Tambopata, Madre de Dios; 29, Manu, Madre de Dios; 30, Oxapampa, Pasco; 31, Ayabaca, Piura; 32, Huancabamba, Piura; 33, Morropon, Piura; 34, Sandia, Puno; 35, Puno, Puno; 36, Moyobamba, San Martin; 37, Lamas, San Martin; 38, Tocache, San Martin. (Adapted from a map available at http://english.freemap.jp/).
(TIF)

S2 Fig Schematic presentation of the coding region of the mannose phosphate isomerase (*mpi*) gene and location of primers for amplification of *mpi* gene fragments [17, 23]. Triangles denote primers used for amplification of each *mpi* gene fragment, and nucleotide positions of each primer are in parentheses. An asterisk at nucleotide position 1,082 shows a polymorphic nucleotide between *L*. *(V*.*) peruviana* and *L*. *(V*.*) braziliensis*.
(TIF)

S3 Fig Direct sequence analysis showing all species-specific polymorphic sites of the *Leishmania mpi* gene fragment. (TIF)

S1 Table "Fragment size of leishmanial mpi gene generated by digestion with selected restriction enzymes". (DOCX)
