# Supplementary figures and images for: Nuclear and kinetoplast DNA analyses reveal genetically complex Leishmania strains with hybrid and mito-nuclear discordance in Peru
Source: PLoS Negl Trop Dis. 2020 Oct 19;14(10):e0008797. doi: 10.1371/journal.pntd.0008797 (PMC7595639; doi:10.1371/journal.pntd.0008797)

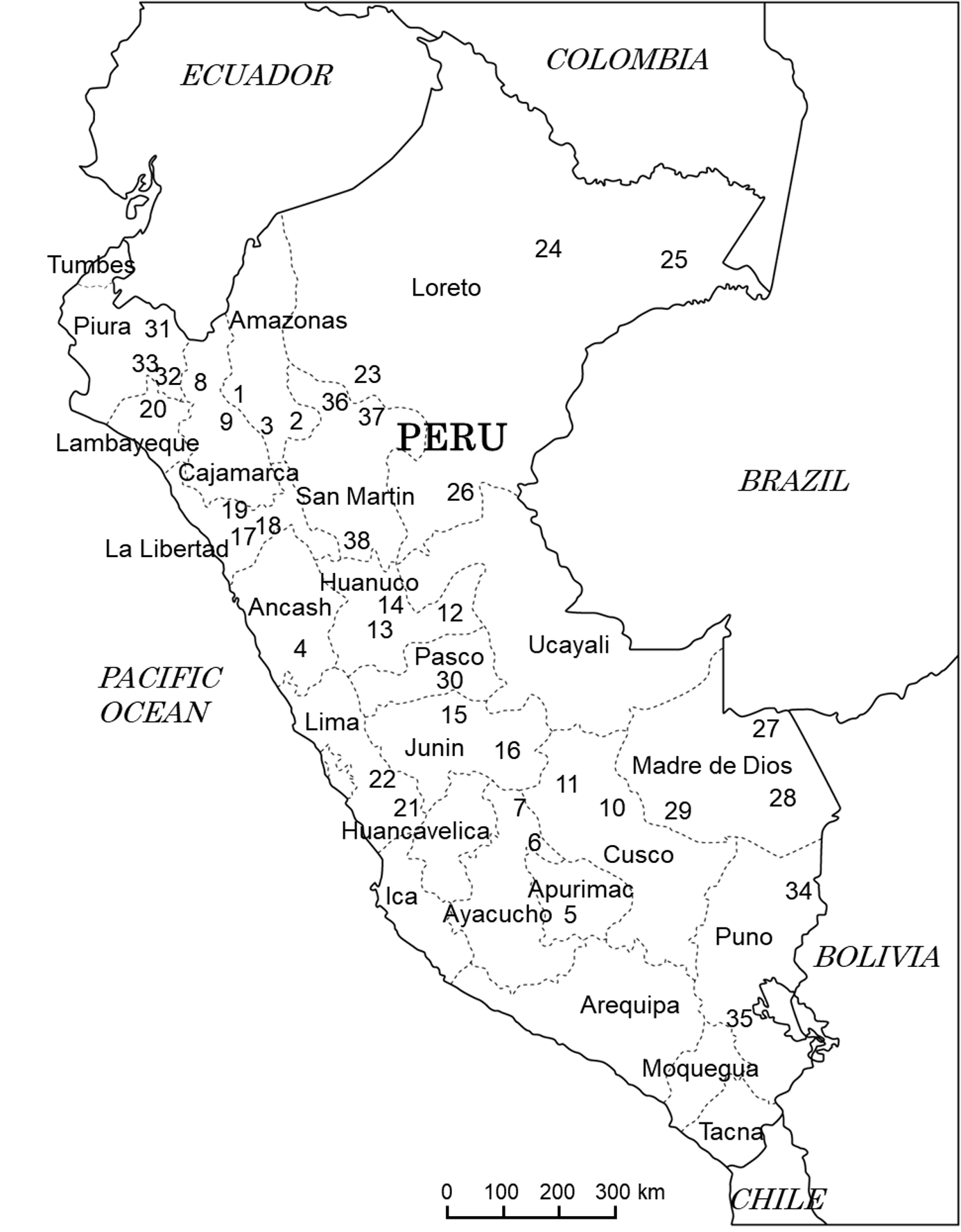

Supplement: S1 Fig — 1, Utcubamba, Amazonas; 2, Rodriguez de Mendoza, Amazonas; 3, Luya, Amazonas; 4, Huarmey, Ancash; 5, Aymaraes, Apurimac; 6, La Mar, Ayacucho; 7, Huanta, Ayacucho; 8, Jaen, Cajamarca; 9, Cutervo, Cajamarca; 10, Calca, Cusco; 11, La Convención, Cusco; 12, Puerto Inca, Huanuco; 13, Huanuco, Huanuco; 14, Leoncio Prado, Huanuco; 15, Chanchamayo, Junin; 16, Satipo, Junin; 17, Otuzco, La Libertad; 18, Santiago de Chuco, La Libertad; 19, Gran Chimú, La Libertad; 20, Lambayeque, Lambayeque; 21, Yauyos, Lima; 22, Huarochirí, Lima; 23, Alto Amazonas, Loreto; 24, Maynas, Loreto; 25, Mariscal Ramón Castilla, Loreto; 26, Ucayali, Loreto; 27, Tahuamanu, Madre de Dios; 28, Tambopata, Madre de Dios; 29, Manu, Madre de Dios; 30, Oxapampa, Pasco; 31, Ayabaca, Piura; 32, Huancabamba, Piura; 33, Morropon, Piura; 34, Sandia, Puno; 35, Puno, Puno; 36, Moyobamba, San Martin; 37, Lamas, San Martin; 38, Tocache, San Martin. (Adapted from a map available at http://english.freemap.jp/). (TIF) [file pntd.0008797.s001.tif]

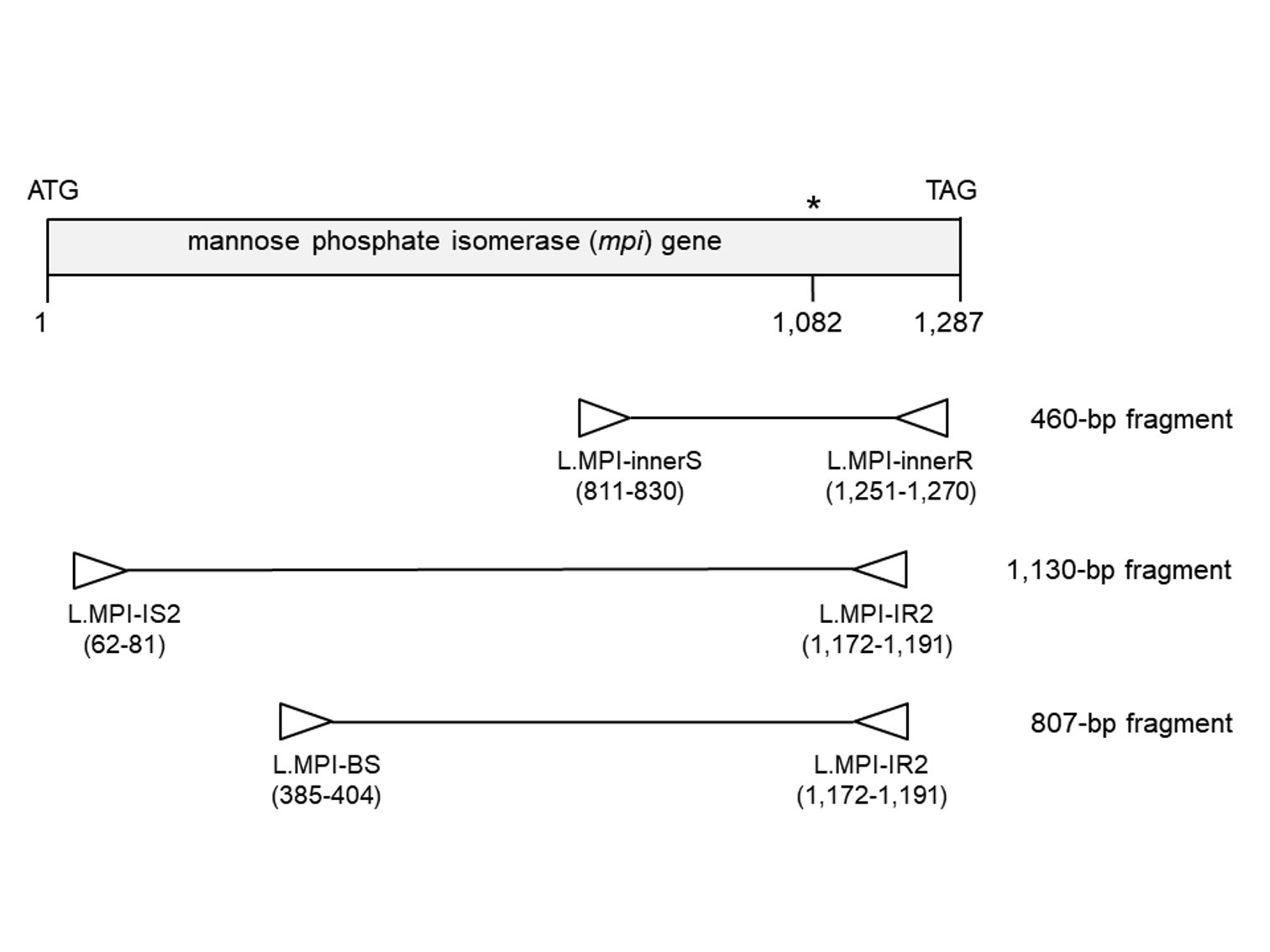

Supplement: S2 Fig — Triangles denote primers used for amplification of each mpi gene fragment, and nucleotide positions of each primer are in parentheses. An asterisk at nucleotide position 1,082 shows a polymorphic nucleotide between L. (V.) peruviana and L. (V.) braziliensis. (TIF) [file pntd.0008797.s002.tif]

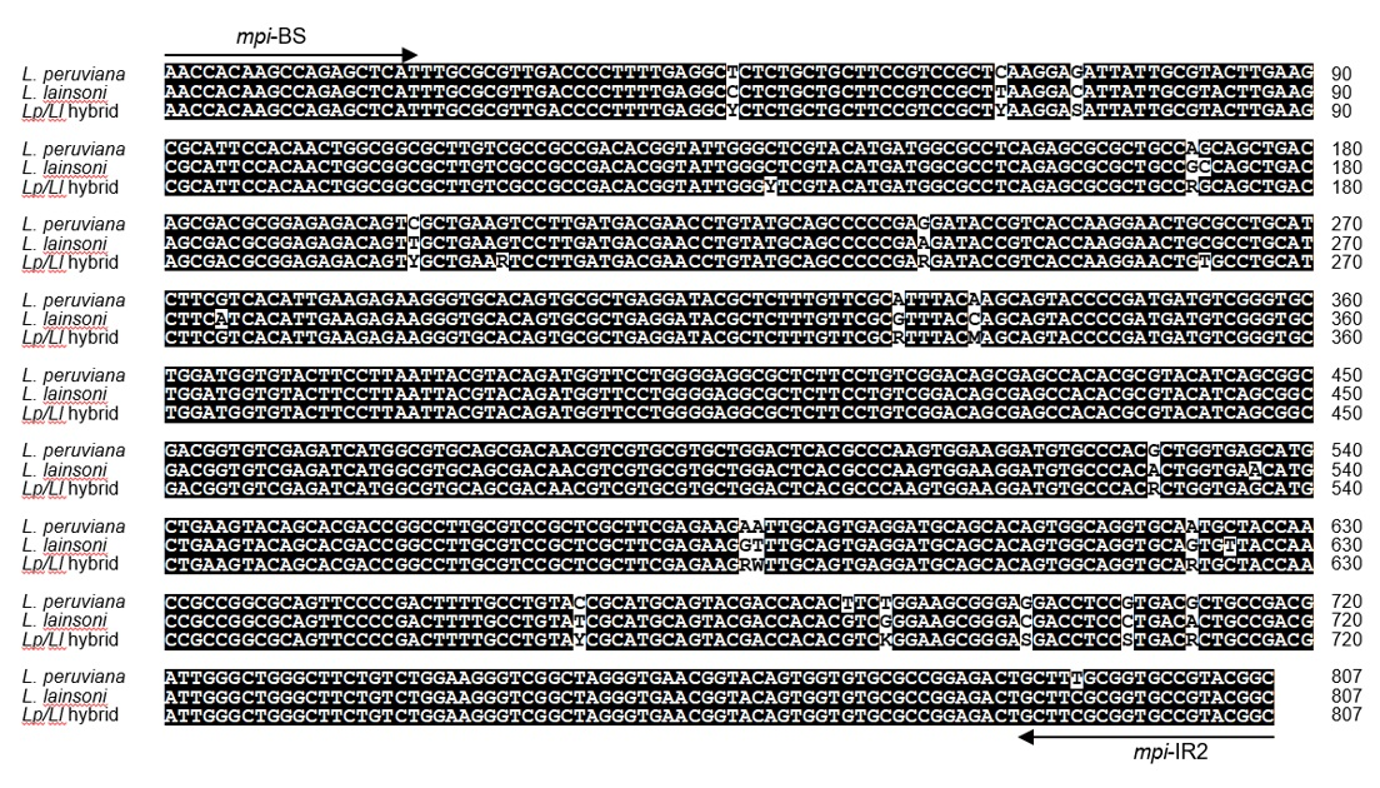

Supplement: S3 Fig — (TIF) [file pntd.0008797.s003.tif]
