# Supplementary material for: Nuclear and kinetoplast DNA analyses reveal genetically complex Leishmania strains with hybrid and mito-nuclear discordance in Peru
Source: PLoS Negl Trop Dis. 2020 Oct 19;14(10):e0008797. doi: 10.1371/journal.pntd.0008797 (PMC7595639; doi:10.1371/journal.pntd.0008797)
Supplement: S1 Table — (DOCX) [file pntd.0008797.s004.docx]

**Supplementary Table**

Table S1. Fragment size of leishmanial *mpi* gene generated by digestion with selected restriction enzymes

| *Leishmania* species | *Hae*III | *Vpa*K11BI | *Bst*XI |
| --- | --- | --- | --- |
| *L. (V.) peruviana* | 560, 247 | 109, 698 | 122, 685 |
| *L. (V.) braziliensis* | 560, 247 | 807 | 122, 685 |
| *L. (V.) guyanensis* | 43, 247, 517 | 807 | 122, 685 |
| *L. (V.) lainsoni* | 43, 247, 517 | 807 | 807 |
| *L. (L.) amazonensis* | 70, 162, 493, 82 | 807 | 807 |
